# The pre-Jurassic Meng-Shaan paleochannel and its control on the accumulation model of Yan 10 reservoir in Central Ordos Basin

**DOI:** 10.1038/s41598-023-42493-4

**Published:** 2023-09-22

**Authors:** Chunling Guo, Jianchao Liu, Guomin Fu, Lijun Chen, Feng Wang, Zhe Huang, Li Qiao, Longjun Wang

**Affiliations:** 1 School of Petroleum Engineering and Environmental Engineering, Yan’an University, Yan’an, 716000 China; 2 School of Earth Science and Resources, Chang’an University, Xi’an, 710054 China; 3 Exploration and Development Technology Research Center, Yanchang Oil Field Co., Ltd., Yan’an, 716000 China; 4 Jingbian Oil Extraction Plant of Yanchang Oil Field Co., Ltd., Yulin, 718500 China; 5 Water Injection Headquarter of Yanchang Oil Field Co., Ltd., Yan’an, 716000 China

**Keywords:** Solid Earth sciences, Sedimentology

## Abstract

In the setting of the increasing density of exploration wells, and the decreasing scale and increasing difficulty of discovering Jurassic paleogeomorphology reservoirs, it is urgent to deepen the fine depiction of pre-Jurassic paleogeomorphology features and to analyze their controlling effect on reservoirs. Based on abundant logging data combined with indoor microscopic observation and experimental testing, the paper applies the theory of reservoir configuration analysis for the first time to the single-channel period division of the pre-Jurassic Meng-Shaan paleochannel which deposited with the braided channel sand bodies. Meanwhile, it reveals the controlling effect of hydrocarbon accumulation and the distribution characteristics of the Yan 10^1^ reservoir as ' paleogeomorphology and sedimentary facies combination' to determine the type, 'migration channel' to determine the distributing range, and 'low-amplitude structure' to determine the trap in the study area, and also the reservoir accumulation modes in different channel ranges have established. In conclusion, the single-channel boundary of the Meng-Shaan paleochannel in different periods controls the path of oil and gas migration, thus controlling the distribution range and reservoir type of the Yan 10 reservoir. Moreover, depending on the production data we derived that it should prefer the composite trap reservoir of the Fuxian period and the Yan 10^2^ period, as well as the structural reservoir on the periphery of the Yan 10^2^ period single-channel for the further exploration of the Yan 10 paleogeomorphology reservoirs within the development range of the Meng-Shaan ancient river. In particular, the lithologic trap reservoirs within the Fuxian period channel and the Meng-Shaan main channel, such as the reservoirs of the 'Source' of the secondary channel type and the paleochannel type, which could as a replacement accumulation model for increasing reserves and production.

## Introduction

At the end of the Late Triassic, the Ordos Basin was affected by the Indosinian movement and uplifted rapidly on a large scale. The top of the Triassic Yanchang Formation was subjected to different degrees of erosion and deformation, forming a paleogeomorphology erosion surface with widespread water systems and longitudinal-horizontal valleys^1,2^, resulting in paleogeomorphology units such as highland denudation areas, low hills, deep valleys, valley plains, and depressions. Among them, the paleochannel units of deep valleys include five major river channel deposits as Gan-Shaan, Qing-xi, Ning-Shaan, Meng-Shaan, and Jin-Shaan ancient rivers^3^. This paleo-erosion surface has a significant control effect on the later deposition and hydrocarbon accumulation^4,5^. The thick coarse clastic rocks of the Fuxian Formation and Yan 10 oil layer were deposited in the erosion valley, and the variegated sand-mudstone interaction layer was deposited on the flood plain, forming one of the most concernment oil-bearing strata in the Ordos Basin, the Jurassic Yan 'an Formation^6,7^. Compared with the Triassic Yanchang Formation ultra-low permeability reservoir, the Jurassic Yan 'an Formation reservoir has the characteristic of shallow drilling depth, large oil-bearing area, thick oil layer, good reservoir physical properties, and high reservoir filling degree^8^. Once the Yan 'an Formation reservoir is produced, the output is several times higher than the oil-bearing layers of the Yanchang Formation, which has important exploration value. The predecessors have carried out a lot of research work on the Yan 'an Formation in the basin and formed the theory of paleogeomorphology controlling reservoir based on the oil exploration mode of 'paleogeomorphology covering channel sand trap'^2^, as well as the single-factor-controlled accumulation mode of river confluence triangle type in Maling oilfield^9^, slope-mouth, mound-mouth, and inter-channel monadnock type in Longdong and Zhenyuan Oilfields^10–12^. However, the discovered reservoirs so far show that some of the reservoirs have been far away from the paleochannels, and extended into the belly of the paleo-highlands. In addition, the chicken-nest-like oil and gas enrichment of the Yan'an Formation, the complex control factors, and the overall distribution pattern are not easy to grasp^13^. It is too difficult to predict reservoirs according to the previous theory, which makes the exploration risk higher.

In recent decades, with the demand for in-depth development of oil and gas reservoirs, the reservoir architecture characterization of sand body superposition and distribution characteristics in sedimentary bodies from the perspective of hierarchical structure has been developed, which is different from the traditional outcrop and sedimentary facies analysis. It provides the necessary theoretical basis and technical support for the efficient and economic development of oil and gas fields^14,15^. Besides, Li et al.^16^ and other researchers believe that the Yan9, Yan8, and Yan7 oil-bearing formations of the Upper Jurassic distributed on the northeast bank of the Meng-Shaan paleochannel, and the Lower Jurassic Fuxian and Yan 10 reservoirs mainly occurred on the southwest bank. However, the exploration practice found that the main producing formation in the east bank of Mong-Shaan paleochannel, such as Xuezhuang of the Dingbian County, Zhouchang area of Wuqi County located in the middle of the Ordos Basin, is the Yan 10 oil-bearing layer^17^. Therefore, in this paper, in order to exclude the cause of differential reservoir enrichment due to pressure differences between the two sides of the valley, we take the Yan10 reservoir in the area of Dingbian-Jingbian-Wuqi on the eastern margin of the Meng-Shaan ancient river as the research object. With the abundant well data in the region, the first attempt is made to apply the idea of hierarchical analysis to dissect the upper-overlay deposition of the pre-Jurassic paleogeomorphology unit, refine and focus on the analysis of the different period channel boundaries of the Meng-Shaan paleochannel, and analyzes its control effect on the accumulation mode of the Yan 10 reservoir, so as to further promote the development of geological theories related to paleogeomorphology reservoirs and provide the necessary theoretical basis for the efficient and economic development of Jurassic oil fields.

## Geological settings

**Figure 1:**
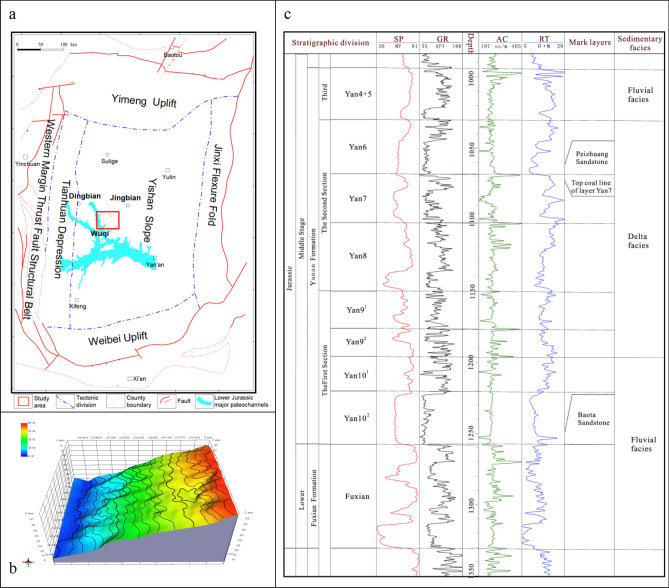
(**a**) Location of the study area, (**b**) structural characteristics of top surface of Yan 10 oil layer and (**c**) stratigraphic division markers and electrical curve characteristics.

The study area is located in the middle of the Yishan slope, a secondary tectonic unit in the Ordos Basin, with simple internal tectonics and only some micro low-amplitude structures. It includes three administrative regions of Dingbian, Jingbian, and Wuqi, with a total area of about 1.8 × 10^3^ km^2^ (Fig. 1a, b). According to the regional stratigraphic correlation study and the characteristics of the rock-electric response of the marker layers, the Jurassic Yan 'an Formation is divided into 4 sections and 10 minor layers from top to bottom^18^. The Yan 1 to Yan 3 layers in the fourth section are deficient within the study area, and the Yan 10 layer is the main producing layer in the study area. According to the cycle of sedimentation, it is divided into two sub-layers, Yan 10^1^ and Yan 10^2^, and Yan 10^1^ sub-layer is the main oil-bearing layer (Fig. 1c). The lithology of the reservoir is mainly blocky medium-grained gray, taupe feldspathic lithic sandstone, and lithic quartz sandstone, which belongs to medium porosity and medium–low permeability reservoir with good physical properties. Due to the influence of the late Triassic Indosinian movement, the Jurassic strata in the study area were developed on the regional angular unconformity denudation surface and the undulating paleo-topography. The Fuxian Formation and the Yan 10 layer of the Jurassic Yan 'an Formation are the fillings of the negative-geomorphic units, which belong to the braided river-filled gully type deposition^19,20^. The exploration practice shows that oil distribution in the study area is mainly controlled by paleogeomorphology, and its reservoir-forming conditions are closely related to the paleogeomorphology units of the Meng-shaan paleochannel. The flow direction of the ancient river determines the direction of the provenance in the study area, i.e., northwestward^21^. In addition, the Meng-Shaan paleochannel belongs to the incised valley, and the exposed strata at the bottom of the valley are the Yanchang Formation Chang 2 oil layer. In terms of Jurassic oil and gas accumulation, it not only shortens the distance from the oil source in the center of the lake basin, but also its thick channel sand body and the unconformity surface at the bottom are the dominant channels for oil and gas migration^11^^,^^22^.

## Methodology

In the paper, we collected logging data for 580 wells, core and test analysis data for 50 wells, and production data for 160 development wells in the study area. Through field outcrop and indoor observations, including core description, casting thin section, scanning electron microscopy, inclusions analysis, and other microscopic observations, as well as combined with the pre-Jurassic paleogeology, lithofacies paleogeography, and sand body distribution characteristics, the pre-Jurassic paleogeomorphology was restored by impression method^6^, and revealed the sedimentary and reservoir characteristics of the Jurassic Yan'an Formation in the study area. Secondly, guided by the classification scheme of fluvial reservoir architecture proposed by Miall^14^, and the principle of 'vertically dividing channel periods and laterally dividing sand body boundaries'^15^, the braided channel deposition on the paleogeomorphology unit of the Meng-Shaan paleochannel in the study area was finely dissected. Furthermore, by comparing the relationship between the single-channel results of the Meng-Shaan ancient river and the distributed features of the Yan 10 reservoir, and analyzing the controlling factors of hydrocarbon accumulation, the accumulation models of the different-period channels have been established. Finally, we classify and summarize the actual exploration data of different accumulation modes, to make the research results more practical, and to better serve the actual production.

## Research process and results

### Pre-Jurassic paleogeomorphology restoration

**Figure 2:**
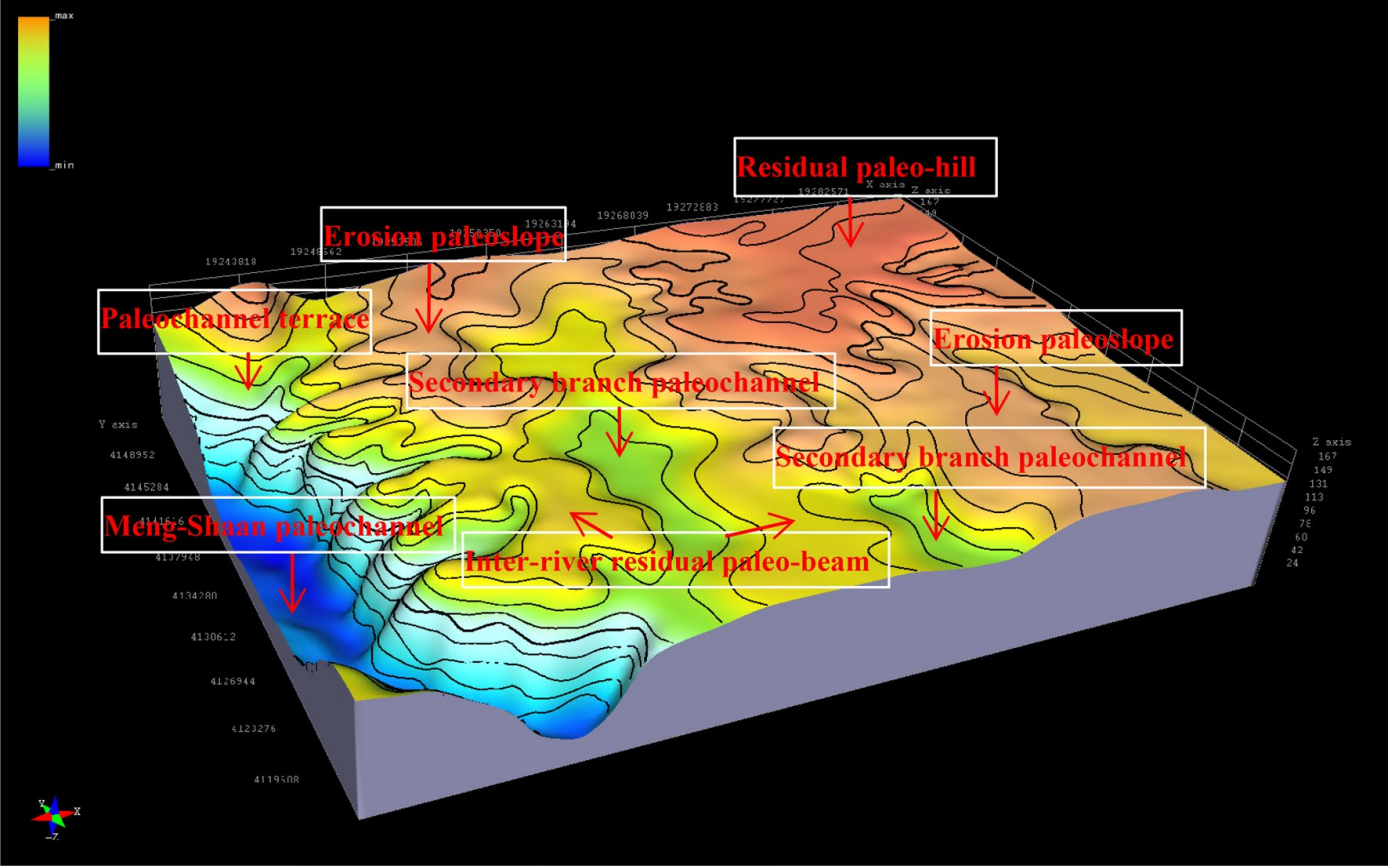
Three-dimensional display of pre-Jurassic paleogeomorphology features in the study area.

Paleogeomorphology is the main controlling factor affecting the development and spatial configuration of sedimentary systems in the early Jurassic^23^, Huang et al^24^, which controls the basic elements of reservoir formation such as reservoir-cap assemblage and oil migration and accumulation in the later reservoirs, thus controlling the formation and distribution of Jurassic oil and gas^25,26^. Therefore, the fine characterization of paleogeomorphology is an important prerequisite for exploring the control of paleogeomorphology on hydrocarbon formation and the hydrocarbon formation law. This paper, based on sedimentary analysis of the pre-Jurassic paleogeology map, lithofacies paleogeography map, stratigraphic sedimentary thickness map, and sand body cumulative thickness map^12^, and according to the principle of impression method^27^^,^^28^, by eroding the thickness of the strata deposited on the paleogeomorphology, that is, the thickness of the filling Yan 10 layer and Fuxian Formation in the study area from thick to thin reflects the paleogeomorphology from low to high, to inverse the undulating morphology of the pre-Jurassic erosion surface. The results show that the paleogeomorphology of the study area includes the Meng-Shaan paleochannel unit with a sedimentary thickness of more than 160 m,the Paleochannel terraces with a sedimentary thickness of 160–140 m,and the Secondary branch paleochannels with a sedimentary thickness of 140–120 m,the Inter-river residual paleo-beams which are the higher terrains between the two undercut valleys with a sediment thickness of 120–100 m,the Erosion paleoslopes with a sediment thickness of 100–80 m and the Residual paleo-hills with a sediment thickness of less than 80 m. There are six types of paleogeomorphology units in Fig. 2, showing a 'west low east high, south gentle north steep ancient erosion surface morphology.

### Sedimentary characteristics research

**Figure 3:**
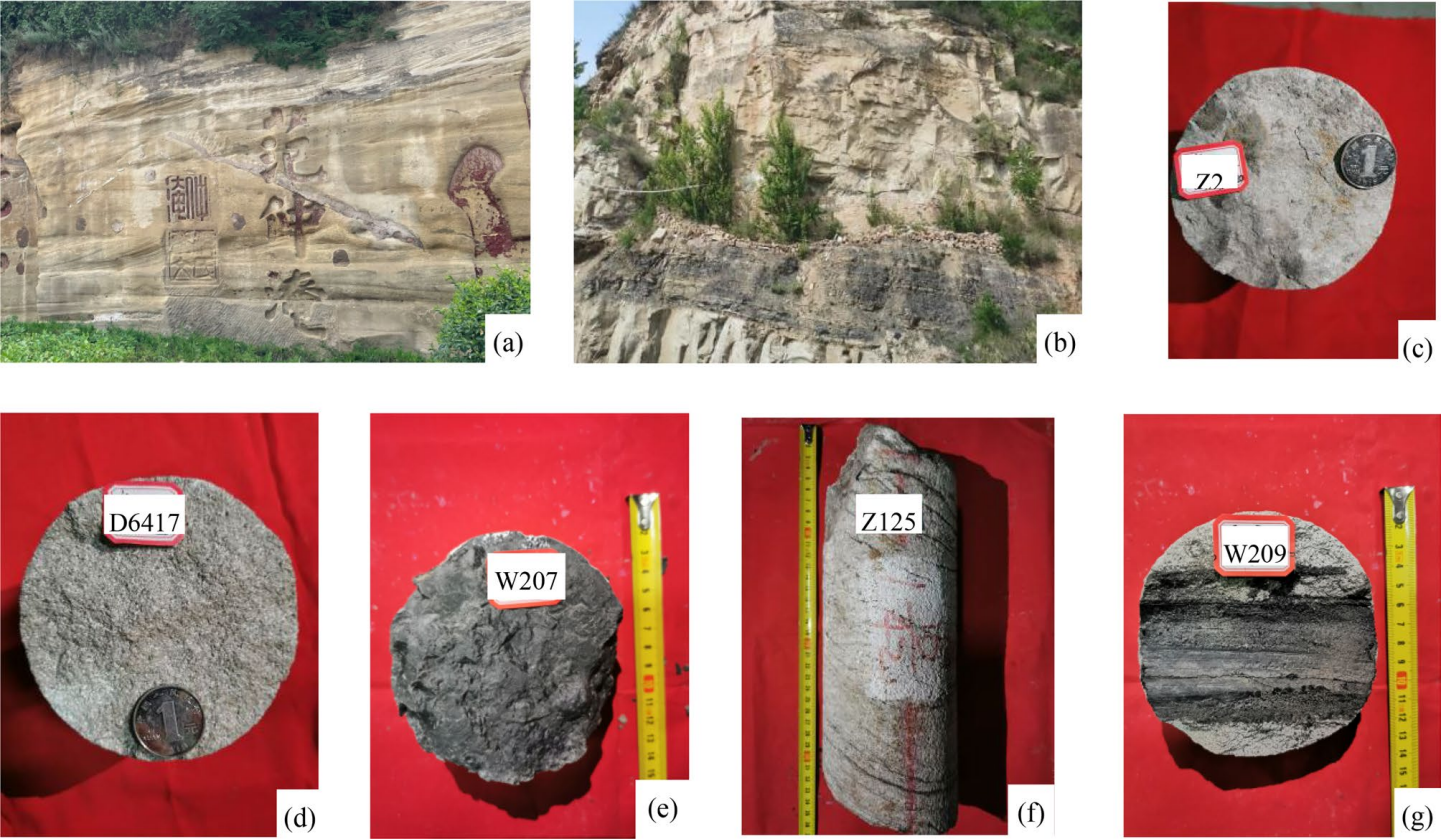
Photographs of the outcrop section and cores showing the sedimentary structural characteristics. (**a**) the thick coarse sandstone in the lower part of Yan 10 layer, with large trough cross-bedding; (**b**) the carbonaceous coal line in the middle of Yan 10 oil formation and the fine sandstone in the upper part; (**c**) bottom conglomerate sedimentation, Yan 10, Z2; (**d**) coarse sandstone of block structure, Yan 10, D6417; (**e**) carbonaceous mudstone, Yan 10, W207; (**f**) tabular cross-bedding, Yan 10, Z125; (**g**) carbonated plant fossil stem, Yan 10, W209.

**Figure 4:**
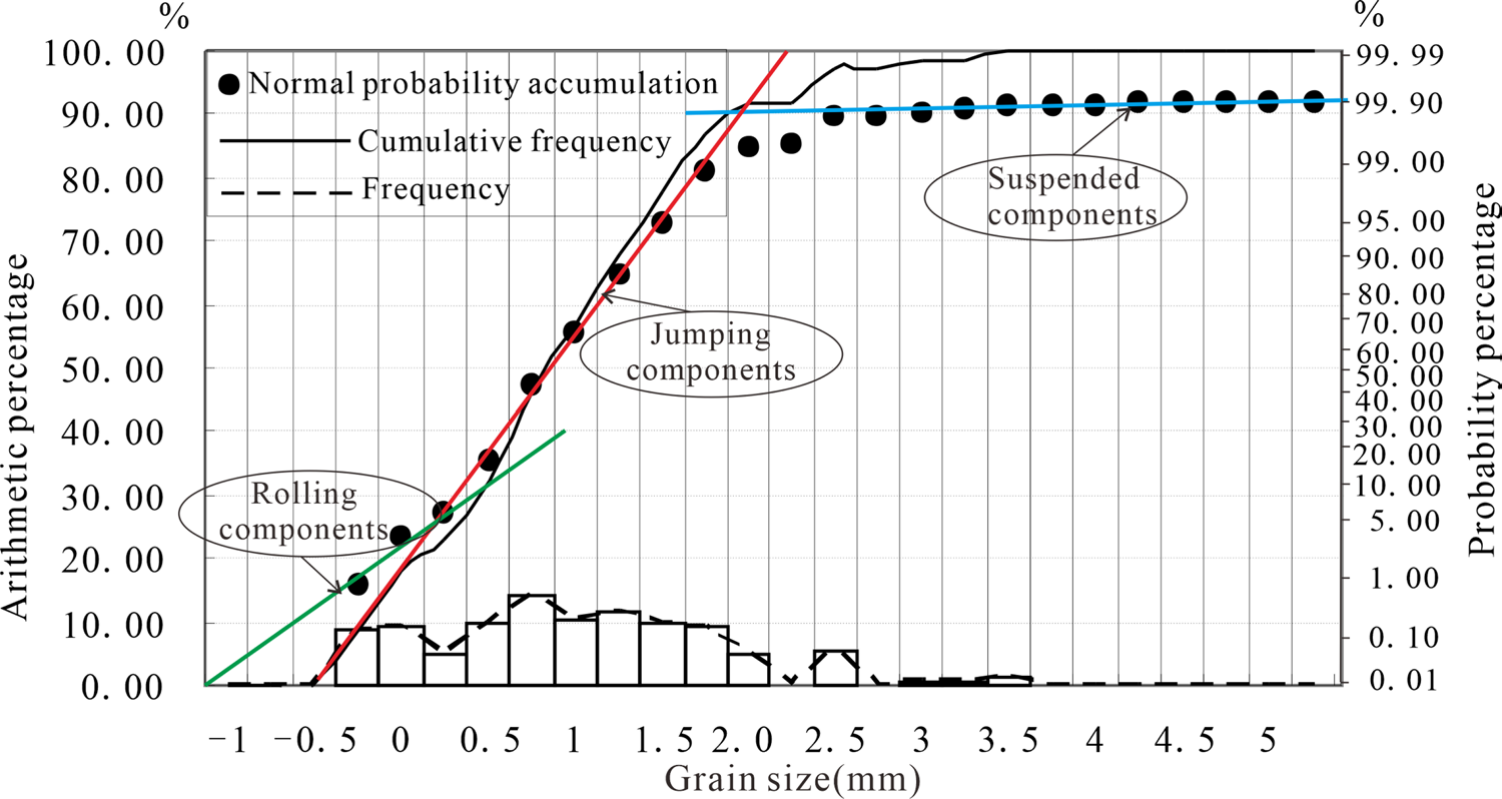
Probability cumulative curve of Yan 10 sandstone grain size, with the characteristics of three-stage type (D6621, sampling depth, 1425.35–1425.52 m) (illustration of legend:1-medium to fine sandstone; 2-fine sandstone; 3-siltstone; 4-mudstone; 5-carbonaceous mudstone; 6-little cross-bedding; 7-tabular cross-bedding; 8-ripple bedding; 9-trough cross-bedding; 10-sand-mud mixed layer).

**Figure 5:**
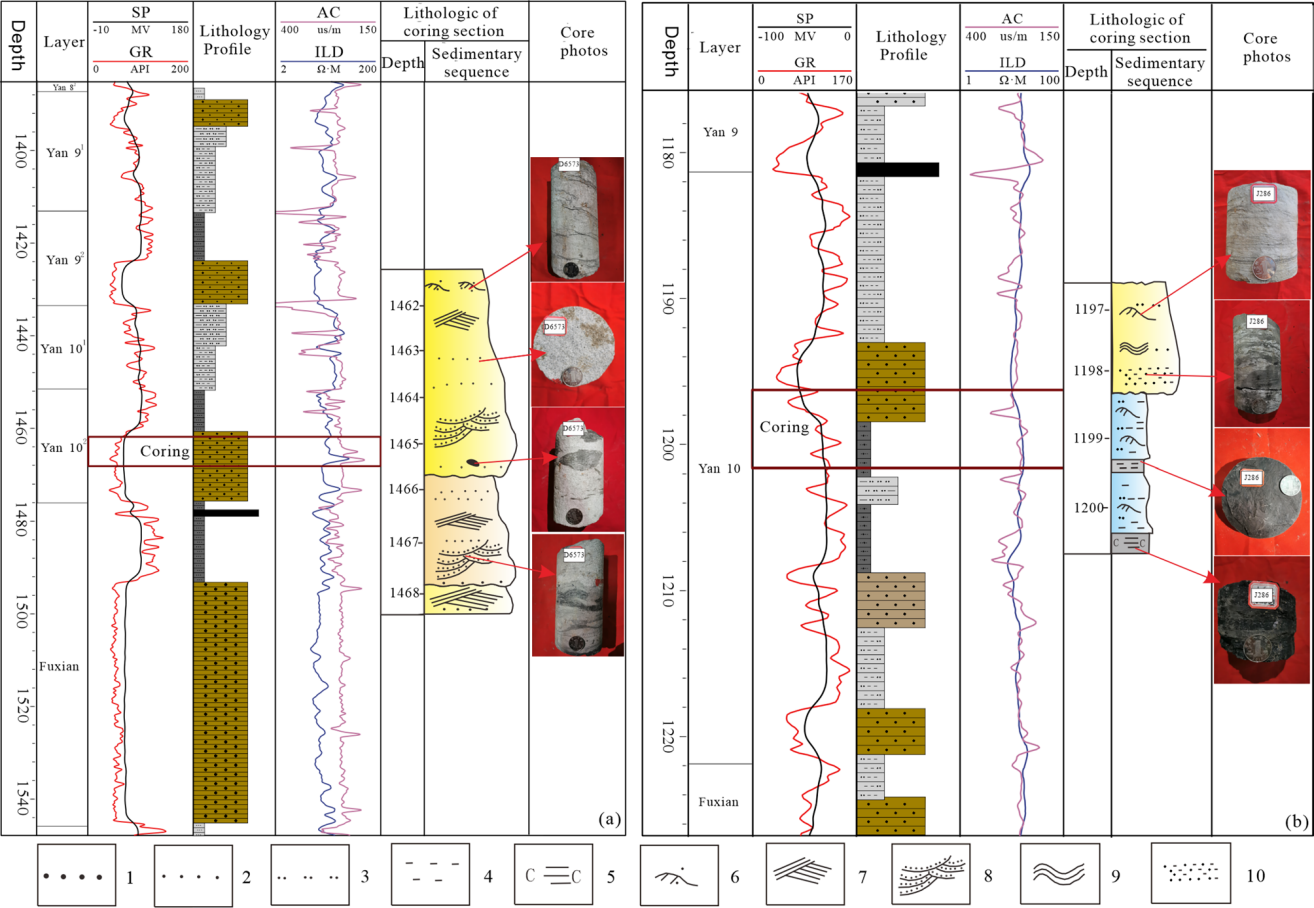
Sedimentary facies map of single well in the Yan 10 layer. (**a**) Channel bar subfacies, D6573; (**b**) floodplain subfacies, J286.

The Jurassic Yan 10 layer and Fuxian Formation developed in the ancient river valley on the top of the Triassic strata. Under the action of erosion and deposition on the valley, the negative topography is gradually filled up^29^. Through the analysis of field outcrops and Jurassic reservoir core data from 50 exploration wells in the study area, the lithology of the Yan 10 layer is dominated by coarse-grained, thick-bedded sandstone (Fig. 3a, d) and sandy gravel (Fig. 3c). The fine-grained sediments are mostly silty mudstone and carbonaceous mudstone (Fig. 3b, e). The sedimentary structure shows large trough cross-bedding, tabular cross-bedding (Fig. 3a, f), and containing carbonized fossil stems or carbonaceous debris (Fig. 3g), etc. Which reflects the high turbidity fluvial depositional environment^30^. In addition, due to the different characteristics of the grain size probability accumulation curve of sandstone formed in different environments, the grain size image analysis results of the thin section in the study area show that the probability curve is three-stage, with rolling components, which are generally deposited by the riverbed underflow (Fig. 4), reflecting the braided river channel environment. Therefore, the Yan 10 oil formation mainly develops fluvial deposits with braided river characteristics. Furthermore, combined with the single well facies logging curve and sedimentary sequence analysis, these sediments include braided channel subfacies (including channel lag deposits and channel sand dam microfacies) and flood plain subfacies (including flood plain and side bar microfacies) (Fig. 5).

**Figure 6:**
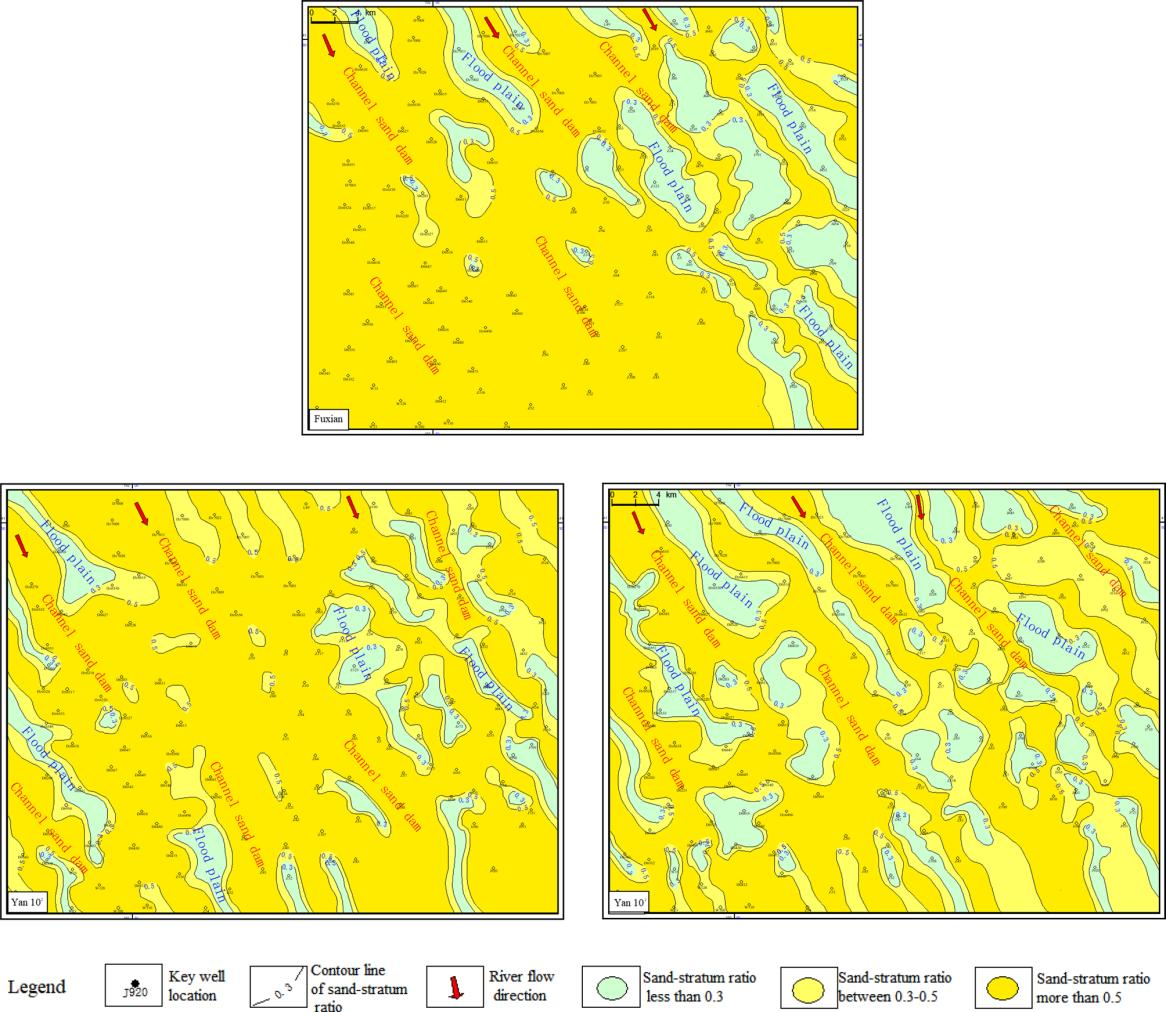
Sedimentary microfacies planar distribution characteristics of the Fuxian Formation, the Yan10^1^, and the Yan10^2^ sub-layer in the study area.

The sedimentary microfacies plane distribution maps of the Fuxian group and each sub-layer of the Yan 10 layer are compiled (Fig. 6). It is obvious that the deposition of the Yan 10 layer in the study area is controlled by the pre-Jurassic paleogeomorphology, and the distribution direction of the main channel is consistent with the Meng-Shaan paleochannel. From the Fuxian Formation to the Yan 10^2^ and Yan 10^1^ sedimentary system, the development has a good inheritance, and the distributary channels further advances, showing a wide and gentle channel in the western part of the study area. The main channel with a ratio of sand-stratum between 0.50 and 0.80, which gradually evolved to the channel sand dam and floodplain braided interweave development from the Fuxian period to the Yan 10^1^ period. The evolution process of this sedimentary system is consistent with the gradual filling of gullies and ravines of the paleogeomorphology.

### Single-channel division of the Meng-Shaan paleochannel overlay deposition

The above sedimentary study shows that the Meng-Shaan paleochannel pre-Jurassic paleogeomorphology unit, located at the western edge of the study area has huge thick braided channel sand bodies imported by the Meng-Shaan ancient river from northwest to southeast overlayed on its negative topography, which provides superior reservoir conditions for the formation of oil reservoirs. Furthermore, the spatial superposition relationship of sand bodies, especially the connectivity between single sand bodies, is crucial for hydrocarbon accumulation. The non-permeable barrier between sand bodies has a considerable influence on oil and gas migration^17^. Therefore, dissecting the inner configuration of the channel thick sand bodies in the Meng-Shaan paleochannel could be finer for exploring the reservoir accumulating law of the hydrocarbons.

**Figure 7:**
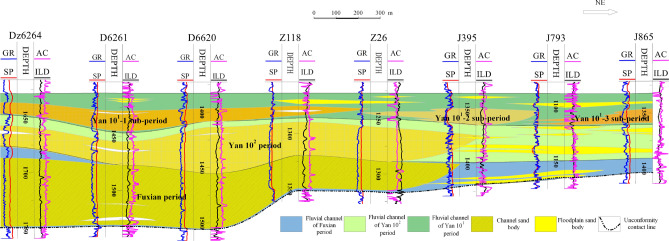
Section of channel period division and its vertical distribution characteristics of the Meng-Shaan paleochannel.

The sedimentary discontinuity surface of fine-grained sedimentation is the main marker of the vertical division of the thick channel sand bodies, and its electrical logging curves such as SP, RT mutation change, or return characteristics are more obvious and easier to identify as the thickness of fine-grained sedimentation becomes larger. Accordingly, by analyzing the characteristics of single-well logging curves and the longitudinal deposition pattern of the river^31^, through the cross-well profiles comparison method, the Meng-Shaan paleochannel overlay deposition was vertically divided into three periods of single-channel sand bodies, namely the Fuxian, Yan 10^2^ and Yan 10^1^ period (Fig. 7). Meanwhile, since several branch channels may exist in the same area during the same period to intersect and stack laterally, as shown in Fig. 6, the distribution of braided branch channels in the Yan 10^1^ layer. Based on the lateral boundary division markers of the channels, such as the elevation difference of the top of the channel sand, the difference in the scale of the channel sand, the distribution of the sedimentary sand bodies and the abandoned channels, and the width and distribution of the single channel sand body^32^, we divide the Yan 10^1^ period of the Meng-Shaan paleochannel into three single sub-channel units (Fig. 7).

**Figure 8:**
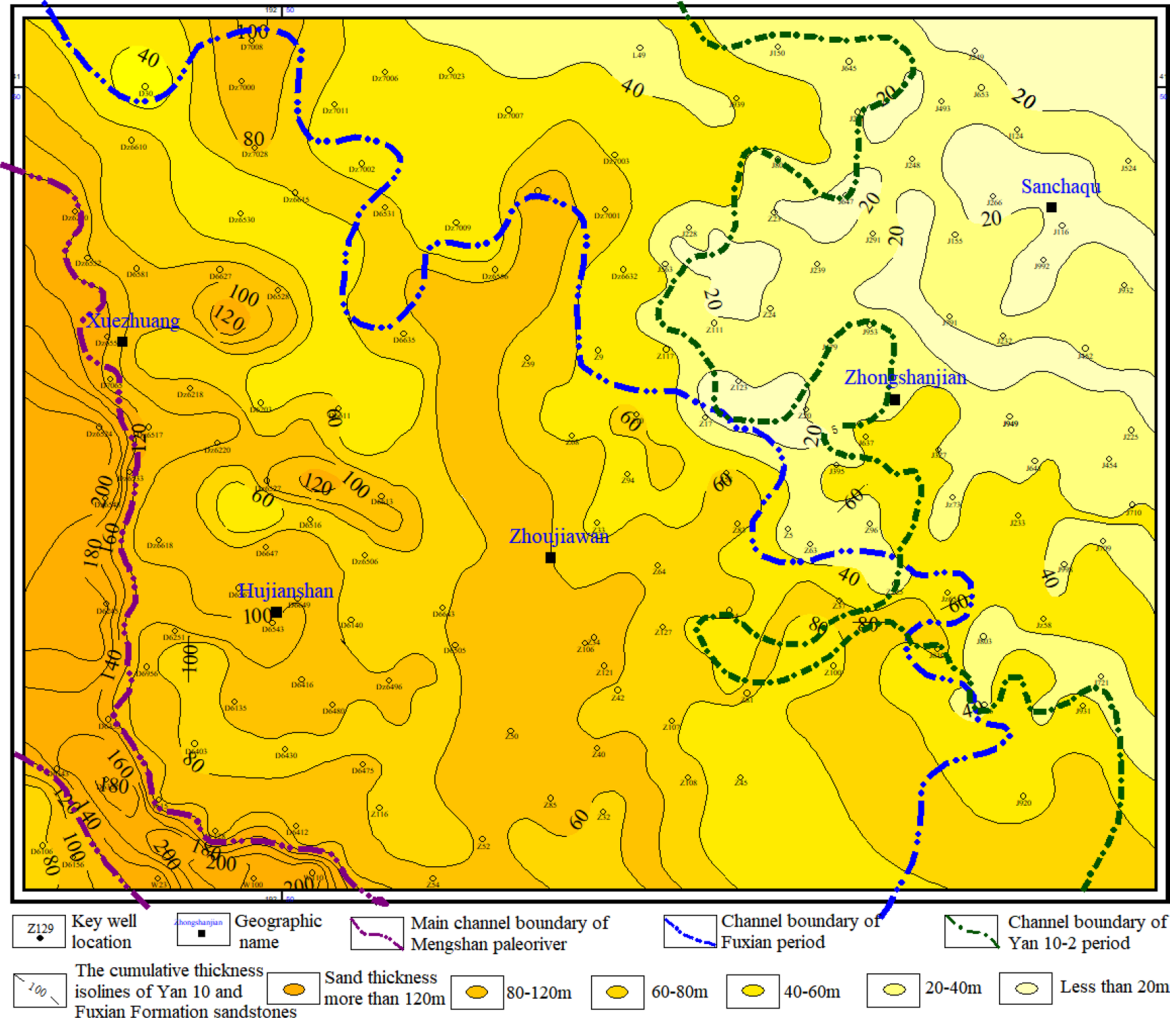
Contour map of the cumulative sand thickness of the Fuxian Formation and Yan 10 layer with the different periods channel boundary line of the Meng-Shaan paleochannel.

Finally, through the comparison and division of the cross-well section of the whole area in the direction of the cutting provenance, it is projected onto the plane map to form two channel boundaries of the Fuxian period and Yan 10^2^ period. In addition, combined with the distribution range of paleogeomorphology and the cumulative thickness contour map of the sandstone from the Yan 10 layer and Fuxian Formation, the main channel boundary of the Meng-Shaan paleochannel has been divided (Fig. 8). From the comprehensive analysis of the paleogeomorphology map and the cumulative sand thickness map, it can be concluded that the main channel boundary of the Meng-Shaan paleochannel located in the Meng-Shaan paleochannel paleogeomorphology unit with huge sedimentary sand bodies filled, where the thickness is greater than 120 m. The channel boundary of the Fuxian period was located in the southwest block of the study area, and the thickness of the sedimentary sand bodies was more than 60 m, which included Meng-Shaan paleochannel, paleochannel terraces, secondary branch paleochannels, inter-river residual paleo-beams, and erosion paleo-slope paleogeomorphology units. The channel width of the Yan 10^2^ period was wider and extended to the northeast of the study area. It was in the range of sedimentary sand body thickness greater than 40 m in the study area, and its boundary was roughly located at the junction of the erosion paleo-slope and the residual paleo-hills paleogeomorphology unit.

## Discussion

### Distribution characteristics and controlling factors of Yan 10 reservoir

#### Yan 10 Reservoir distribution characteristics

**Figure 9:**
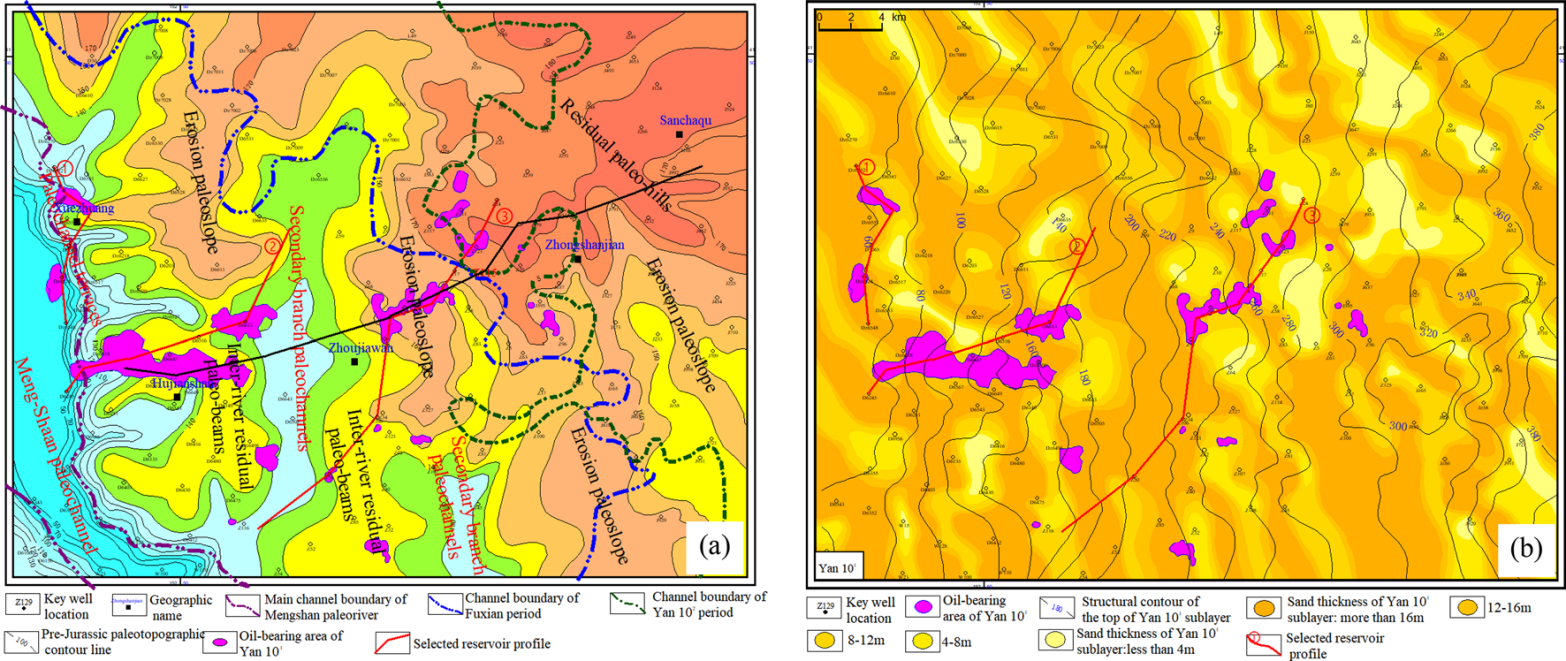
**(a) **Diagram of Yan 10^1^ reservoirs superimposed with the pre-Jurassic paleogeomorphology and the boundary of different periods of the Meng-Shaan paleochannel, and **(b)** the Yan 10^1^ reservoirs superimposed with the sand thickness and top-surface structure of Yan 10^1^ layer.

**Figure 10:**
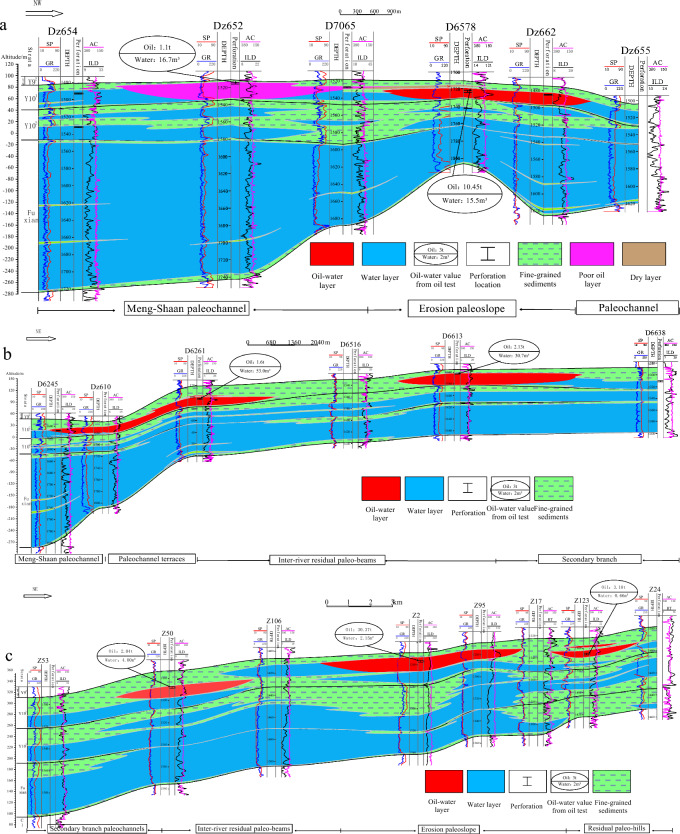
Three typical reservoir cross-sections of the Fuxian Formation and the Yan 10 oil-bearing layer and its corresponding plane positions are shown in Fig. 9 red lines.

Based on the results of the period division of the Meng-Shaan paleochannel overlay deposition, we superimposed the proven Yan 10^1^ reservoirs in the study area with the results of the paleogeomorphology restoration, and the boundary of the different periods of the Meng-Shaan paleochannel (Fig. 9a). And also, superimposed the proven Yan 10^1^ reservoirs to the thickness distribution of the sand body and the top structure of the Yan 10^1^ sub-layer (Fig. 9b). Three typical reservoir profiles of the Fuxian Formation and Yan 10 layer were selected for anatomy (Fig. 10). Whose purpose was to analyze the control effect of Jurassic paleogeomorphology background on the Yan 10^1^ reservoir and the accumulation mode of the Yan 10^1^ reservoir.

1. Dz6548–Dz6552 well reservoir cross-section (Fig. 10a), located in the western margin of the study area, crossed the Meng-Shaan paleochannel, Paleochannel terraces, and the Erosion paleoslope paleogeomorphology units, in which the oil-bearing well Dz6524 is located in the main channel of the Meng-Shaan paleochannel. The reservoir type is the thick sand body of the braided channel, the thickness can reach 18 m. Its structural position is relatively high, which is a sandstone lens reservoir. Wells Dz6578 and Dz6629 are located in the slope mouth of the Erosion paleoslope at the edge of the deep-incised paleochannel (that is, the slope mouth of the steep paleoslope, calculated according to the triangular geometric relationship, the slope angle is about 1.1°). The nose uplift structure formed a trap, crude oil migrated upward through the sand bodies of the paleochannel and the unconformity erosion surface, and the braided channel sand bodies formed a good reservoir condition, which is a structural lithologic reservoir.
2. The reservoir cross-section of well D6245–D6638 (Fig. 10b), along the direction of the vertical provenance, crosses four paleogeomorphology units, namely, the Meng-Shaan Paleochannel, Paleochannel terraces, Secondary branch paleochannels, and Inter-river residual paleo-beams. The oil-bearing wells Dz6102 and D6261 well of profiles locate in the mound mouth part of the Paleochannel terraces, which have a low amplitude structure of the nose uplift, channel sand reservoir conditions, and rich oil source near the deep-incised valley, and the effective thickness of the oil layer is 8 m, and 15 m, respectively. Which belongs to a structural lithologic reservoir. Well D6613 is located in the ‘Source’ of the secondary branch paleochannel, with the condition of nearing the oil source and the lithological up-dip physical deterioration, which forms a lithological up-dip pinch-out reservoir.
3. The reservoir profile of the Z53–Z24 well (Fig. 10c), which is spreading in the northeast direction, spans four paleogeomorphology units, including the Secondary branch paleochannel, Inter-river residual paleo-beam, Erosion paleoslope, and the Residual paleo-hill. The Z50 oil well locates at the ‘Source’ of the secondary channel near the edge of the Inter-river residual paleo-beam. The oil-bearing area of the block where the well locates is very small, but it produces 2.04 t of oil per day and 4.00 m^3^ of water per day, with high production per unit area, which is a lithologic reservoir. The Z2 and Z95 oil wells are located in the Erosion paleoslope paleogeomorphology unit near the Secondary branch paleochannel, which has a large oil-bearing area and high production per well. The daily oil production of the Z2 well is 30.37 t, and the daily water production is 2.15 m^3^. Its reservoir-forming conditions are superior. The reservoir is distributed along the rows of nose uplift, the sand body is thicker and less disturbed by the bottom water, and the top cover is well capped, it is a structural lithologic reservoir. The Z123 well is located at the Residual paleo-hill paleogeomorphology unit at the edge of the channel boundary of the Yan 10^2^ period. Crude oil migrates along the unconformity surface at the bottom of the channel sand bodies to the structural high point to form a structural reservoir.

Through the detailed analysis of the reservoir cross-sections and combined with the superimposed plane map (Fig. 9), it can be derived that the Jurassic Yan 10^1^ reservoir in the study area has the following characteristics:

1. The proven reservoirs are distributed in the Inter-river residual paleo-beam, Erosion paleoslope, Residual paleo-hill, Paleochannel terrace, and Meng-Shaan paleochannel paleogeomorphic unit in order of the reservoir area from large to small, in which the erosion paleoslope has the highest yield per unit area;
2. According to the location relationship between reservoir distribution and paleogeomorphology unit, combined with the thickness of reservoir sand bodies and structural characteristics, the reservoirs in the study area can be categorized into seven types: paleochannel, ’Source’of the secondary channel, residual paleo-beam, mound mouth, slope mouth, paleoslope, and paleo-hill type;
3. Different reservoir types have different distribution ranges. The paleochannel type of the Yan 10^1^ reservoir is distributed inside the main channel of the Fuxian period, and the paleo-hill type reservoir is distributed near the outer boundary of the channel boundary of the Yan 10^2^ period. While the other types of reservoirs are inside the channel boundary of the Fuxian period. There is no Yan 10^1^ reservoir in the northeast of the study area far from the channel boundary of the Yan 10^2^ period.

#### Analysis of accumulation conditions and controlling factors of Yan 10 reservoir

**Figure 11:**
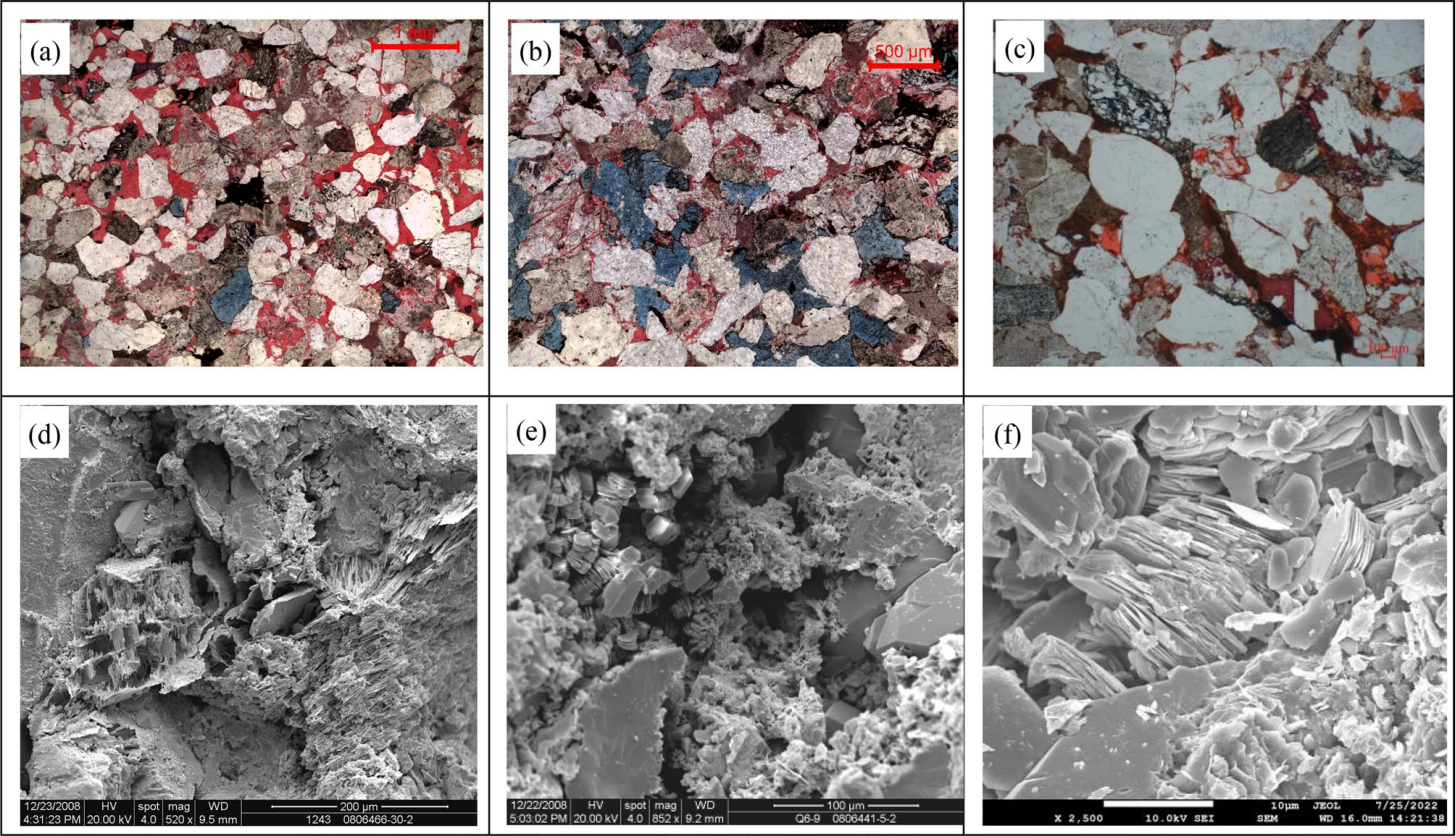
Yan 10 reservoir space type in the study area. (**a**) Intergranular pores, feldspar dissolved pores, Z10-41 well; (**b**) feldspar dissolution, Z75; (**c**) debris dissolution pore, residual intergranular pore, D6621; (**d**) debris dissolution, J515; (**e**) illite and kaolinite filling intergranular pores, J517 ; (**f**) kaolinite intercrystalline pore, Z129.

To clarify the main oil source in the study area, we selected 10 mudstone samples from Yan 10 of Yan 'an Formation in the area for oil shale testing and analysis. In comparison with the Chang 7 source rocks in the center of the lake basin in terms of organic matter abundance, type, maturity, and biomarker characteristics, it is considered that the oil source of the Yan 10 reservoir in the study area is the Chang 7 source rock in the center of the lake basin, which is consistent with the research conclusions of the Yang et al.,^33^. The incised valley of the Meng-Shaan paleochannel in the western margin of the study area has its bottom channel sandstone directly overlying the Chang 2 oil layer of the Yanchang Formation, which greatly shortens the distance from the oil source at the bottom, and the negative geomorphic units such as the secondary branch paleochannel and the branch-ditch cut the top layer of the Yanchang Formation, which creates favorable windows for the study area to preferentially capture oil from the bottom up. Under the combined action of regional strata pressure, slope pressure at the edge of the ancient river, and buoyancy, hydrocarbon migrates laterally from the bottom of the Meng-Shaan paleochannel to the high point of the Jingbian erosion paleoslope through the unconformity surface and the superimposed sand body migration channel^16^. At the same time, it migrates vertically through fractures and connected sand bodies to accumulate in favorable reservoir sand bodies of the Yan 10 layer^34^. Petrographic casting thin sections and scanning electron microscopy analysis shows that the main pore types of Yan 10 layer in the study area are intergranular pores, feldspar dissolved pores and a small amount of debris dissolved pores, intergranular dissolved pores and intercrystalline pores (Fig. 11). Among them, intergranular pores and dissolved pores are the main reservoir space. According to the statistics of the physical property analysis data of the Yan 10 layer of 50 wells in the study area and adjacent areas, it is concluded that the effective high-quality reservoirs of the Yan 10 layer are mainly controlled by the pre-Jurassic paleogeomorphology, with different paleogeomorphic units having different pore types, pore structures, and porosity and permeability. The statistical results show that intergranular pores and dissolution pores are well developed and have good porosity–permeability in the Meng-Shaan paleochannel, paleochannel terraces, and inter-river residual paleo-beam paleogeomorphic units. In contrast, the secondary branch paleochannel, erosion paleoslope, and residual paleo-hill have slightly poorer conditions (Table 1). Furthermore, the maturity and migration of the Chang 7 source rocks in the Yanchang Formation began in the Cretaceous, which was later than or at the same time as the Yanshan tectonic movement. The spatial and temporal configuration relationship of tectonic formed and hydrocarbon transport is most conducive to the formation and preservation of the Yan 10^1^ reservoir in the study area^6^^,^^35^.

**Table 1:** Comparison of microscopic characteristics of reservoirs in different paleogeomorphological units of Yan 10 layer in the study area

Layer	Paleogeomorphological units	Intergranular pores (%)	Dissolution pores (%)	Average porosity (%)	Average permeability (mD)
Yan 10	Meng-Shaan paleochannel	6.75	1.95	15.3	20.8
	Paleochannel terrace	8.84	2.98	14.5	16.7
	Inter-river residual paleo-beam	5.86	2.77	14.1	13.6
	Secondary branch paleochannel	6.03	1.64	13.8	17.2
	Erosion paleoslope	4.52	1.72	13.5	13.1
	Residual paleo-hill	3.03	1.10	12.9	12.6

Through the above anatomy of the typical oil reservoir profiles and analysis of the the basic conditions of hydrocarbon accumulation, the favorable conditions for the pre-jurassic palaeogeomorphology to control the enrichment and accumulation of Yan 10 oil and gas can be summarized as follows: (1) Deep-cut valleys in paleogeomorphology are a necessary guarantee for connecting oil sources and ensuring upward migration of oil and gas; (2) unconformity erosion surfaces and superimposed sand bodies in eroded paleochannels are the main pathways for oil and gas migration; (3) channel sand bodies under the control of paleogeomorphology are favorable reservoirs for the Yan 10 reservoir; (4) different compacting structures under the background of paleogeomorphology provide preferential directions and good traps for oil and gas enrichment; (5) the combination of reservoir and cap rocks and alternating groundwater flow under the control of paleogeomorphology are effective preservation conditions to prevent oil and gas from escaping; (6) the paleogeomorphology controls the distribution and type of Yan 10 oil reservoir.

**Figure 12:**
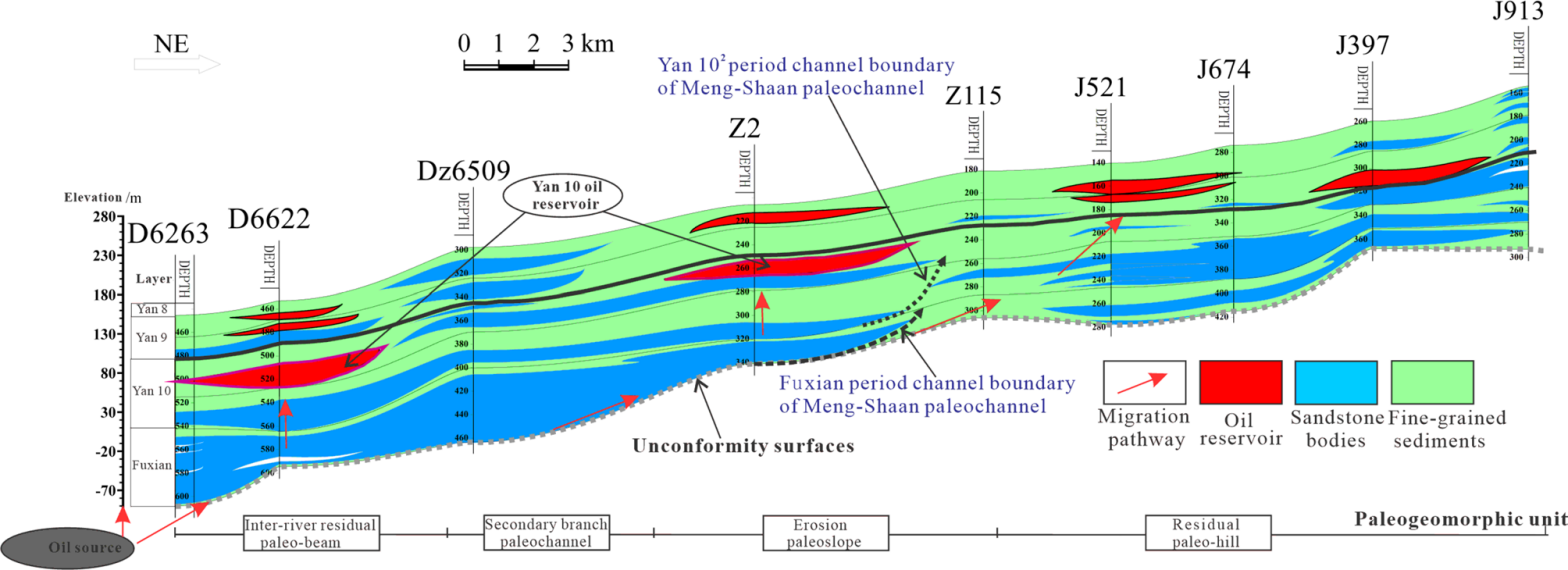
The reservoir profile is used to analyze the relationship between the channel boundary of the Meng-Shaan paleochannel in different periods and the oil migration path of the Yan 10 reservior.

Analysis of the relationship between reservoir distribution and channel boundaries in different periods of the Meng-Shaan Paleochannel shows that the spatial distribution of oil reservoirs is closely related to the hydrocarbon migration pathway(Fig. 9a). Further anatomy of the NE-trending reservoir profile across the Fuxian period and Yan 10^2^ period of the Meng-Shaan paleochannel in the study area (see Fig. 9a, the black line is the location of the profile), it can be seen that the Yan 10 oil wells D6622 and Z2 are located in the Yan 10^2^ period of the river. Which oil migration path is dominated by the vertical migration of the channel sand body and the lateral migration of the Fuxian bottom unconformity surface, forming the residual paleo-beam type—structural lithologic reservoir and paleoslope type—structural lithologic reservoir. The oil migration path of the J521 and J397 wells, which are located at the outer boundary of the channel of the Fuxian period and Yan10^2^ period, is mainly lateral migration of the unconformity surface migration channel, and the oil-bearing layer is higher than that of Yan10 layer, without Yan10 oil reservoir enrichment (Fig. 12). In addition, the Yan 10 oil reservoir in the main channel of the Meng-Shaan paleochannel is dominated by the vertical migration of crude oil from the Chang 7 of the Yanchang Formation at the bottom through the dominant migration channel of the thick sand body in the paleochannel. Therefore, it can be inferred that the architecture of sedimentary sand bodies in the pre-Jurassic Meng-Shaan paleochannel in the study area controls the migration path of the Yan 10 oil and gas accumulation. As a result, it also controls the distribution range and reservoir types of the Yan10 oil reservoir.

In addition, the west-dipping monoclinic structure of the Yan 10 layer top surface formed in the Mesozoic Yanshan stage with rows of nose-uplift structures formed by late local differential compaction. Most of the proven Yan10^1^ reservoirs and oil production wells in the study area are closely related to these nose-uplift structural highs(Fig. 9b). In summary, the main controlling factors for the distribution of the Yan 10^1^ reservoir in the study area are not only controlled by a single paleogeomorphology or paleogeomorphology and sedimentary facies combination^12,22,28^, but by a combination of multiple factors which could be summarized as 'paleogeomorphology and sedimentary facies combination' to determine the type, 'migration channel' to determine the distribute range, and 'structural high point' to determine the trap.

### Genesis mechanism and accumulation model of oil reservoir

The study area is located on the northeast bank of the Jurassic Meng-Shaan paleochannel. The Yan 10^1^ reservoirs distribute as far as the Zhongshanjian area, which is nearly 100 km away from the lake shoreline where the main hydrocarbon-generating strata of the Yanchang Formation developed, and oil fields have been found in the Chang 6 and Chang 2 layers of the Yanchang Formation at the lower strata of the Jurassic Yan’an Formation^36,37^. The ability of hydrocarbon to be transported and accumulated into reservoirs over such long distances is controlled by the oil and gas migration channel and dynamic genetic mechanism. An exception to the laterally and vertically connected sand bodies and the fractures involved in the migration and accumulation of oil^38,39^, the top erosion surface of the Yanchang Formation^40,41^, as well as the regional monoclinic stratigraphic pressure in the study area and the pressure generated by the slope of the northeast bank of the Meng-Shaan paleochannel, the resultant force of the two points to the north-east direction. At the same time, driven by the bottom water, the power of oil and gas migration from the Meng-Shaan ancient deep-incised valley to the high point of the paleoslope is greatly increased^16^. Moreover, the secondary paleochannels are developed in this area, and sand bodies are well connected, so oil and gas under the action of a strong driving force along the secondary channel can not only migrate to Yan 10 oil layer but also continue to move up to the Yan 9 layer and the upper strata of the Yan’an Formation.

**Figure 13:**
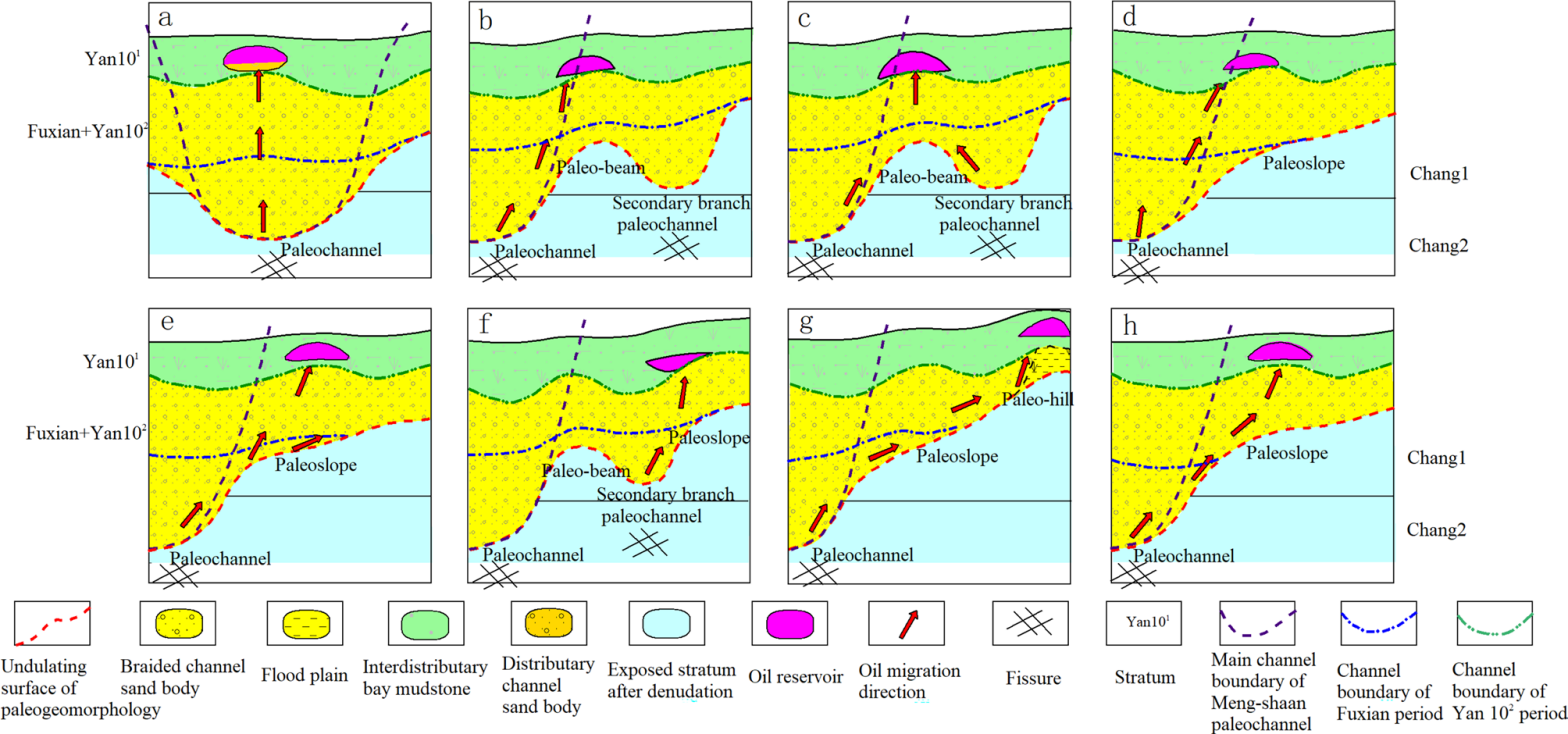
Schematic diagram of reservoir formation mode and genesis mechanism of Yan 10^1^ reservoir.

Overview of the previous studies, the Yan 10^1^ reservoirs are restricted by the basic conditions of oil source, trap, sand bodies distribution characteristics, overlying sedimentary environment, groundwater flow alternation, and so on^24,28^. While paleogeomorphology characteristics, local differential compaction structures on the top surface of the Yan 10^1^ layer, and oil and gas migration channels are the main controlling factors for accumulating for different types of reservoirs in the study area. Moreover, the hydrocarbon accumulation patterns are different within the channel of different periods in the Meng-Shaan paleochannel. Therefore, according to the combination of the trap genesis and the comprehensive control effect of multiple factors, the Yan 10^1^ reservoir in this area could be divided into eight different accumulation models (Fig. 13).

#### Accumulation models in the main channel of Meng-Shaan paleochannel

##### (A) Paleochannel type—lithologic reservoir

The main channel of the Meng-Shaan paleochannel is filled with thick braided channel sand bodies, and the connected sand bodies could form dominant migration channels to migrate crude oil upward captured from deep valleys. The Yan 10^1^ sub-layer has low permeability and high variability due to special depositional conditions and post-formation changes, and good permeability sand bodies exist within large low-permeability sand bodies, forming a typical lithologic trap of sandstone lens blocked by the tight sandstone, such as the Dz6524 well block in the study area.

#### Accumulation models in the Fuxian period of Meng-Shaan paleochannel

##### (B) Mound mouth type—structural lithologic reservoir

In the channel of the Fuxian period, the connected sand bodies and unconformable surfaces are the dominant channels for oil and gas migration. The mound mouth is a sand-rich area located in the tectonic high part of the Paleochannel terrace near a deep-incised valley or located in inter-river residual paleo-beam near the secondary branch paleochannel paleogeomorphology unit, which is the first location reached by oil and gas transportation. The reservoir's physical properties are good due to the repeated swing of the bottom water, and it is easy to form a structural-lithologic trap, such as the Dz6497 and Z57 well blocks in the study area.

##### (C) Residual paleo-beam type—structural lithologic reservoir

The reservoir of the residual paleo-beam type is distributed in the channel of the Fuxian period of the Meng-Shaan paleochannel. It is located in the higher flat roof between two incised valleys and is the dominant pointing location for the oil and gas accumulating that is transported upward along the valley edge on both sides. Low amplitude anticline structures are superimposed with braided channel sand dams to form a structural lithologic reservoir, such as the D6261 and Z129 well blocks in the study area.

##### (D) Slope mouth type—structural lithologic reservoir

Slope mouth type reservoirs are similar to Mound mouth type reservoirs, which are distributed in the Fuxian period channel of the Meng-Shaan paleochannel as well. It is usually divided into two cases. One is distributed in the Paleo-terrace position near the edge of the deep-incised valley, and located at the front edge of the steep Paleoslope paleogeomorphology unit. It preferentially captures oil sources from the valley and accumulates in the high parts of the structure, such as the Dz6629 well block in the study area. The other distribute in the tectonically high part of the leading edge of the gentle Paleoslope near the Secondary branch paleochannel, such as the 10–35 well block. The braided channel sand dam is apt to form a structural lithologic reservoir.

##### (E) Paleoslope type—structural lithologic reservoir

It is distributed in the paleogeomorphology unit Paleoslope within the Fuxian period channel of the Meng-Shaan paleochannel, which integrates the advantageous reservoir formation conditions such as hydrocarbon migration, sedimentary facies belt, low amplitude structure, and relative stagnation of groundwater flow, etc. The structural lithologic traps formed under this accumulation mode, such as the Z94 well block in the study area.

##### (F) 'Source' of the secondary channel type—lithologic reservoir

The concept of ''Source' of the secondary channel' proposed by Li Fengjie et al.^16^ refers to that when the secondary branch ditch developed at the end of the slope near the valley extends to the high point of the slope, the depth of the branch ditch becomes shallower, and the sand body gradually becomes thinner in the upward direction, as if the channel traces back to the source. In the process of oil and gas migration along the valley, in the case of lithology up-dip thinning or pinch-out, the connectivity becomes poor and accumulates in situ, forming lithologic reservoirs, such as D6613 and Z50 well blocks in the study area.

#### Accumulation model at the periphery of the Yan 10^2^ period channel boundary of Meng-Shaan paleochannel

##### (G) Paleo-hill type—structural reservoir

Paleo-hills distribute at the periphery of the channel boundary of the 10^2^ period of the Meng-Shaan paleochannel, which benefits from the lateral migration channel of the unconformity surface at the bottom of the Fuxian Formation, the oil source is migrated from the bottom of the valley to the sand-rich sedimentary facies belt of the paleo-hills and finally accumulated in the high part of the structure. The structure is the primary controlling factor of the accumulation model, such as the Z123 well block in the study area.

#### Accumulation model between the Fuxian period channel and the Yan 10^2^ period channel of Meng-Shaan paleochannel

##### (H) Paleoslope type II—structural lithologic reservoir

In the study area, between the Fuxian period channel and the Yan 10^2^ period channel, the migration of oil and gas is mainly through the erosion surface at the bottom of the Fuxian-period channel laterally to the thick sand bodies of the Yan 10^2^ period channel and then migrate through the thick sand bodies upward to the sedimentary faces of the sand dam of the Yan 10^1^ period braided channel, forming a paleoslope type II structural lithologic reservoir in the high part of the structure, such as the Z6 and 52–56 well blocks in the study area (by the way, named for this which is purpose to distinguish it from the paleoslope type structural lithologic reservoir in the channel of the Fuxian period).

In a word, the reservoirs in the main channel of the Meng-Shaan paleochannel are dominated by the paleochannel-type lithologic accumulation model, and the reservoir accumulation models in the channel of the Fuxian period are dominated by the composite traps, such as the mound mouth type, slope mouth type, residual paleo-beam type, paleoslope type structural lithologic reservoirs, and 'Source' of the secondary channel type lithologic reservoir accumulation mode. While outside the channel boundary of the Yan 10^2^ period, which is dominated by the paleo-hill structural reservoir accumulation model.

Ultimately, the statistical results of reservoir enrichment degree (Table 2) show that the various trap-genetic reservoirs in different periods of the Meng-Shaan paleochannel have different enrichment degrees. The composite-trap reservoirs of structural-lithologic reservoirs of the Fuxian period of the Meng-Shaan paleochannel have the characteristics of large quantity, large area, large reserves, and high enrichment degree. The average geological reserves per unit area are 47.16 × 10^4^ t/km^2^. The structural trap-genetic reservoirs locate outside the channel boundary of the Yan 10^2^ period are of medium degree of enrichment, with an average geological reserve of 39.36 × 10^4^ t/km^2^ per unit area; while the lithologic reservoirs within the main channel of the Meng-Shaan paleochannel are relatively few in number, small in area, small in reserves, and poor in the degree of enrichment, with an average geological reserve of 38.93 × 10^4^ t/km^2^ per unit area. All in all, the geological reserves per unit area classified by the accumulation mode, in descending order are paleoslope type, residual paleo-beam type, paleoslope type II, slope mouth type, mound mouth type, paleo-hill type, 'source' of the secondary channel type and paleochannel type.

**Table 2:** Statistics of the proven reserves of different accumulation models and different trap-genesis reservoirs in the study area.

Accumulation model	Plane computing well area	Oil-bearing area (km^2^)	Geological reserves (10^4^ t)	Geological reserves per unit area of different accumulation models (10^4^t/km^2^)	Trap genesis	Geological reserves of different traps (10^4^t)	Geological reserves per unit area (10^4^t/km^2^)
Paleochannel	Dz6524	1.99	75.19	37.78	Lithologic	311.41	38.93
‘Source’ of the secondary channel	D6613	5.61	226.84	39.30			
	Z50	0.40	9.38				
Residual paleo-beam	Z129	1.32	76.87	58.23	Structural lithologic	1491.81	47.16
Residual paleo-beam, mound mouth	D6261	16.91	697.79	41.26			
Mound mouth	Dz6497	1.90	59.16	33.89			
	Z57	0.17	10.99				
Slope mouth	Dz6629	2.36	93.08	42.28			
	10-35	0.57	30.79				
Paleoslope	Z94	7.00	448.70	63.60			
	126-4	0.14	5.38				
Paleoslope II	52-56	0.14	3.27	54.80			
	Z6	1.12	65.78				
Paleo-hill	Z102	0.95	29.33	39.36	Structural	183.83	39.36
	Z89	1.60	54.40				
	Z69	1.95	93.01				
	44843	0.17	7.09				

Therefore, we suggest that in the further exploration of the Yan 10 paleogeomorphology reservoir within the development range of the Meng-Shaan ancient river, it should be based on the composite trap reservoir of the Fuxian period channel of the Meng-Shaan paleochannel, such as the residual paleo-beam type, the paleoslope type, the slope mouth type, and the mound mouth type reservoirs. As well as the paleohill-type structural reservoir on the periphery of the Yan 10^2^ period channel. Meanwhile, the 'Source' of the secondary channel type and paleochannel type reservoirs can be carried out within a reasonable economic evaluation range for the need for increasing reserves and production.

## Conclusion

1. The pre-Jurassic paleogeomorphology of the study area includes six different geomorphic units, of which the Meng-Shaan paleochannel unit, overlay it deposited by the braided channel sand bodies, that are divided into the Fuxian period, the Yan 10^2^ period, the Yan 10^1^ period single channel, and the main channel of the Meng-Shaan paleochannel.
2. Depending on the location of paleogeomorphology units, the Yan 10^1^ reservoirs are classified into seven types, and various reservoir types distribute in different periods of the Meng-Shaan paleochannel. Furthermore, the controlling factors of hydrocarbon accumulation and the distribution characteristics of the Yan 10^1^ reservoir are described as 'paleogeomorphology and sedimentary facies combination' to determine the type, 'migration channel' to determine the distributing range, and 'low-amplitude structure' to determine the trap in the study area.
3. The migration paths of oil and gas are different in the channel range of different periods of the Meng-Shaan paleochannel, which leads to different accumulation modes of the Yan 10 reservoir. Among them, the geological reserves enrichment of the paleoslope type, residual paleo-beam type, paleoslope II type, slope mouth type, mound mouth type belonging to composite trap reservoir within the Fuxian period channel and Yan 10^2^ period channel of the Meng-Shaan paleochannel. The paleo-hill type structural reservoir at the periphery of the Yan 10^2^ period channel is medium. Meanwhile, the lithologic trap reservoirs which conclude the 'Source' of the secondary channel type within the Fuxian period channel and the paleochannel type of the Meng-Shaan main channel, the degree of geological reserves enrichment is the third, which could be used as a strategic replacement accumulation model for increasing reserves and production

## Data Availability

The data used to support the findings of this study are available from the corresponding authors upon request.
